# Whole Body Periodic Acceleration Is an Effective Therapy to Ameliorate Muscular Dystrophy in *mdx* Mice

**DOI:** 10.1371/journal.pone.0106590

**Published:** 2014-09-02

**Authors:** Francisco Altamirano, Claudio F. Perez, Min Liu, Jeffrey Widrick, Elisabeth R. Barton, Paul D. Allen, Jose A. Adams, Jose R. Lopez

**Affiliations:** 1 Department of Molecular Biosciences, School of Veterinary Medicine, University of California Davis, Davis, California, United States of America; 2 Department of Anesthesiology Perioperative and Pain Medicine, Brigham & Women’s Hospital, Harvard Medical School, Boston, Massachusetts, United States of America; 3 Department of Physiology, Perleman School of Medicine, University of Pennsylvania, Philadelphia, Pennsylvania, United States of America; 4 Division of Genetics and Program in Genomics, Boston Children’s Hospital, Harvard Medical School, Boston, Massachusetts, United States of America; 5 Anatomy and Cell Biology, School of Dental Medicine, University of Pennsylvania, Philadelphia, Pennsylvania, United States of America; 6 Division of Neonatology, Mount Sinai Medical Center, Miami, Florida, United States of America; Medical College of Georgia, United States of America

## Abstract

Duchenne muscular dystrophy (DMD) is a genetic disorder caused by the absence of dystrophin in both skeletal and cardiac muscles. This leads to severe muscle degeneration, and dilated cardiomyopathy that produces patient death, which in most cases occurs before the end of the second decade. Several lines of evidence have shown that modulators of nitric oxide (NO) pathway can improve skeletal muscle and cardiac function in the *mdx* mouse, a mouse model for DMD. Whole body periodic acceleration (pGz) is produced by applying sinusoidal motion to supine humans and in standing conscious rodents in a headward-footward direction using a motion platform. It adds small pulses as a function of movement frequency to the circulation thereby increasing pulsatile shear stress to the vascular endothelium, which in turn increases production of NO. In this study, we examined the potential therapeutic properties of pGz for the treatment of skeletal muscle pathology observed in the *mdx* mouse. We found that pGz (480 cpm, 8 days, 1 hr per day) decreased intracellular Ca^2+^ and Na^+^ overload, diminished serum levels of creatine kinase (CK) and reduced intracellular accumulation of Evans Blue. Furthermore, pGz increased muscle force generation and expression of both utrophin and the carboxy-terminal PDZ ligand of nNOS (CAPON). Likewise, pGz (120 cpm, 12 h) applied *in vitro* to skeletal muscle myotubes reduced Ca^2+^ and Na^+^ overload, diminished abnormal sarcolemmal Ca^2+^ entry and increased phosphorylation of endothelial NOS. Overall, this study provides new insights into the potential therapeutic efficacy of pGz as a non-invasive and non-pharmacological approach for the treatment of DMD patients through activation of the NO pathway.

## Introduction

Duchenne muscular dystrophy (DMD) is a X-linked recessive and progressive muscle disease caused by failure to express sarcolemmal protein dystrophin [1], . DMD is the most common muscular dystrophy observed in children. The estimated worldwide incidence of DMD is approximately 1∶3500 male live births [3]. Dystrophin is a key component of the dystrophin glycoprotein complex (DGC), which links the cytoskeleton of the muscle fibers to the extracellular matrix [1], [2], [4]. In the absence of dystrophin, DGC is functionally impaired such that mechanical stress associated with contraction leads to the degeneration of muscle fibers [5], [6]. It is now well established that the lack of dystrophin expression in skeletal and cardiac *mdx* muscles leads to several secondary processes including inflammation, alteration of intracellular ion homeostasis, chronic degeneration and regeneration and necrosis/apoptosis of muscle fibers, metabolic alterations and interstitial fibrosis all of which exacerbate the progression of DMD [7].

Cumulative evidence suggests that in addition to its mechanical function as a molecular scaffold, dystrophin plays an important signaling role in both cardiac and skeletal muscles [8]. Thus, the absence of dystrophin is associated with intracellular Ca^2+^ and Na^+^ overload in DMD patients [9] and *mdx* mice [10], [11], alterations in transient receptor potential channel function (TRPC) [12] and activation of several Ca^2+^-dependent intracellular signaling pathways in skeletal muscle [10], [11], [13].

Although the genetic defect responsible for DMD was identified decades ago [4], currently there is no effective treatment available for this devastating disease. Administration of corticosteroids and related drugs to diminish inflammation in DMD [14] have limited efficacy along with significant side effects, such as respiratory muscle weakness, hypoxemia, fatigue, and hypoventilation during sleep [15]–[17]. The need for new treatments have led investigators to focus on multiple therapeutic strategies such as gene and cell based therapies designed to bypass the mutation (exon skipping) or to replace the missing gene and/or dystrophin protein, which have achieved varying degrees of success [18], [19]. Although such treatments are in clinical trials, new pharmacological strategies appear promising and can circumvent many of the difficulties obstructing gene and cell based therapies [20]. In general, the new pharmacological strategies aim to decrease inflammation, reduce the intracellular Ca^2+^ overload, enhance NO production by providing NO precursors, administer NO donors, or phosphodiesterase type-5A (PDE5A) inhibitors [20]–[24] and/or upregulating utrophin, a compensatory protein whose molecular structure is similar to dystrophin. Thus, there is need for a successful approach that enables patients to survive, improve the quality of life, and thus take advantage of gene therapies when they eventually become available.

pGz is a non-invasive, drug-free approach to enhancing NO pathways, which is produced by applying sinusoidal motion to supine humans and in standing conscious rodents in a headward-footward direction using a motion platform that adds small pulses to the circulation, thereby increasing pulsatile shear stress to the vascular endothelium [25]. Shear stress and pulsatile shear represents the tangential frictional force and axial forces exerted on the luminal walls of the vascular system by the blood flow and are potent regulators of endothelial and neuronal nitric oxide synthase expression (eNOS and nNOS. also referred to as constitutive NOS or cNOS), and of NO production *in vitro* and *in vivo*.

The aim of the current study was to explore the potential therapeutic effect of pGz in the *mdx* mouse model of DMD. We found that pGz treatment caused significant decrease of intracellular Ca^2+^ and Na^+^ overload, reduced sarcolemmal Ca^2+^ entry, diminished serum CK levels, and reduced intracellular accumulation of Evans Blue. Furthermore, pGz improved muscle force generation and up-regulation of both utrophin and the carboxy-terminal PDZ ligand of nNOS (CAPON). These effects were also associated with transient increase in phosphorylation of eNOS. Overall, this study provides new insights about the potential therapeutic utility of pGz to treat patients suffering from DMD.

## Materials and Methods

### Myotube cultures

Primary myoblasts were isolated from hind limbs muscles from wild type (*wt*) C57BL/10 and *mdx* male mice as described previously [10]. Myotubes were differentiated in differentiation media (low glucose DMEM, 4% horse serum) for 3 days and then subjected to pGz inside a humidified incubator at 37°C, 5% CO_2_ and 10% O_2_. Control dishes with non-treated myotubes were maintained at the same incubator. To study the participation of cNOS in pGz signaling pathway we treated myotubes with or without 1 mM N-(G)-nitro-L- arginine methyl ester (L-NAME, Sigma-Aldrich, MO, USA).

### Animals

Male 6-week old C57BL/10 and *mdx* mice were obtained from the Jackson Laboratory (ME, USA). All animals were housed at 23°C with 12 h light-dark cycle and maintained with standard mouse chow and water *ad-libitum*.

### pGz *in vivo* and *in vitro*

The *in vivo* pGz treatment was performed on unanesthetized restrained 6-week old *wt* and *mdx* mice using a reciprocal platform (Scilogex, SK-180-Pro, CT, USA) set at a frequency of 480 cpm, Gz±0.3 m/sec^2^, over a one-hour daily for 8 days. After pGz treatment, animals were returned to their cages in the animal facility. All experiments were carried out 4 h after the last pGz treatment. At the conclusion of the treatment, mice were driven to the laboratory installation to perform the experiments described bellow. The *in vitro* pGz treatment was carried out in *wt* and *mdx* myotubes placed in an incubator (10% O_2_ and 5% CO_2_ at 37°C) and mounted in a reciprocal platform (described above) set at a frequency of 120 cpm for 12 h. Untreated myotubes were kept in the same incubator.

### Ca^2+^ and Na^+^ selective microelectrodes

Double/barreled Ca^2+^-selective and Na^+^-selective microelectrodes were prepared as described previously [26]. The Ca^2+^ ionophore II-ETH 129 or the Na^+^ Ionophore I-ETH 227 (both Fluka Sigma-Aldrich, MO, USA) were used to back-fill the Ca^2+^ and Na^+^-selective microelectrodes, respectively. Each ion-selective microelectrode was individually calibrated as described previously [26]. After making measurements of intracellular resting Ca^2+^ ([Ca^2+^]_i_) and resting Na^+^ concentration ([Na^+^]_i_) all microelectrodes were recalibrated, and if the two calibration curves did not agree within 3 mV, data from that microelectrode were discarded.

### Recording of [Ca^2+^]_i_ and [Na^+^]_i_ in muscle fibers and cultured myotubes

Measurements of [Ca^2+^]_i_, and [Na^+^]_i_, were performed both *in vivo,* in anesthetized (ketamine 100/xylazine 5 mg/kg) *wt* and *mdx* mice muscle fibers and *in vitro* on differentiated *wt* and *mdx* myotubes. For *in vivo* measurements, once the animal was anesthetized, a small incision was made in the skin, the muscle fascia was removed and the superficial fibers of *Vastus lateralis* (left leg) were exposed. A rectal temperature probe was placed, connected to a low noise heating system for maintaining animal body temperature during experimental procedures (WPI-ATC1000, FL, USA). The superficial muscle fibers were perfused with imaging solution (in mM: 140 NaCl, 5 KCl, 2.5 CaCl_2_, 1 MgSO_4_, 5 glucose, and 10 Hepes/Tris, pH 7.4) and were impaled with either the Ca^2+^ or Na^+^ double-barreled microelectrode. The membrane potential signal (V_m_), the Ca^2+^ potential (V_CaE_) and the Na^+^ potential (V_NaE_) were recorded via a high impedance amplifier (WPI Duo 773 electrometer WPI, FL, USA). The potential from the V_m_ barrel (3 M KCl) was subtracted electronically from V_CaE_ or V_NaE_, to produce a differential Ca^2+^-specific (V_Ca_) or Na^+^ specific (V_Na_) potential that represents [Ca^2+^]_i_, or [Na^+^]_i_ based on an experimental calibration curve performed with the same electrode. V_m_, V_Ca_ and V_Na_ were filtered with a low pass filter (30–50 KHz) to improve the signal-to noise ratio and stored in a computer for further analysis. For *in vitro* studies, cells were maintained in imaging solution and impaled with either a double-barreled Ca^2+^- or Na^+^- selective microelectrode and the procedures to obtain the specific potential for Ca^2+^ (V_Ca_) or Na^+^ (V_Na_) were identical to that described above.

### Forelimb grip strength test

Forelimb grip strength was assessed by means of a grip strength meter (Columbus Instruments, OH, USA). *Wt* and *mdx* mice were lifted over the baseplate by the tail so that its forepaws were allowed to grasp onto the steel grid. The mouse was then gently pulled back by the tail until its grip was released. Mice were tested 5 times, with one-minute interval between trials. The three highest measured values were averaged to calculate the grip strength, which was normalized by the body weight in grams.

### *In situ* muscle function measurements

Mice were deeply anesthetized via intraperitoneal (i.p.) injection of ketamine-xylazine mixture (80 and 10 mg/kg) and carefully monitored throughout the experiment. Additional doses were administered as needed to ensure no reflex response to toe pinch. The distal tendon of the *Tibialis Anterior* (TA) muscle was dissected free from surrounding tissue, individually tied with 4.0 braided surgical silk, and then cut at the most distal end. The sciatic nerve was exposed, and all its branches were cut except for the common peroneal nerve. The foot was secured to a platform, and the knee was immobilized using a stainless steel pin. The TA tendon was attached to the lever arm of a 305B dual-mode servomotor transducer (Aurora Scientific, ON, Canada). TA muscle contractions were then elicited by stimulating the distal part of the sciatic nerve via bipolar electrodes, using supramaximal square-wave pulses of 0.02 ms (701A stimulator; Aurora Scientific). Data acquisition and control of the servomotors were conducted using a LabVIEW-based DMC program (version 5.202; Aurora Scientific, ON, Canada). The muscle length was measured using digital calipers based on well-defined anatomical landmarks. The optimal muscle length (L_o_) was determined by incrementally stretching the muscle using micromanipulators until the maximum isometric twitch force was achieved. Three maximum isometric tetanic forces (P_o_) were determined using a train of 150-Hz, 500-ms supramaximal electrical pulses at the L_o_ in the muscle, and the highest P_o_ was recorded. A 2-minute resting period was allowed between each tetanic contraction. The normalized tetanic specific force (N/g) was calculated by dividing P_o_ by the muscle weight.

### Serum Creatine Kinase determinations

Blood samples were obtained from anesthetized *wt* and *mdx* mice by cardiac puncture [11]. Briefly, blood was collected in sterile 1.5 mL eppendorf tube, allowed to clot in ice for 30 min and then centrifuged at 3000 rpm for 10 minutes. Creatine kinase (CK) levels were determined using the UV-kinetic method (Teco Diagnostics, CA, USA) according to the manufacturers instructions. ΔAbsorbance/min were used to calculate CK enzymatic activity and the results were expressed as International Kilo Units per liter (KUI/L).

### Evans Blue uptake

To assess muscle damage, Evans Blue dye (EBD) was used as a marker of permeabilized or damaged muscle fibers. EBD was dissolved in PBS (10 mg/mL) and sterilized by filtration through 0.2 µm filters. Dystrophic *mdx* mice with or without 8 days of pGz treatment were i.p. injected with 0.5 mg dye per 10 g body weight (n = 2 per group). Six hours later, mice were euthanized, the skin was removed, and the animals were photographed and inspected for dye uptake into skeletal muscles, indicated by blue coloration.

### Western blot protein expression analysis

Gastrocnemius muscles were dissected and minced with a pair of scissors and then homogenized with an electric homogenizer (LabGEN 7b, Cole-Parmer, IL, USA) in modified RIPA buffer (150 mM NaCl, 50 mM Tris pH = 7.4, 1% Triton X-100, 0.5% Na deoxycholate, 0.1% SDS, 5 mM EDTA, 2 mM EGTA, 1X Roche Complete Protease Inhibitor and 1x Roche PhosSTOP). Myotubes were pGz-treated, quickly washed with ice-cold PBS, and lysed with modified RIPA buffer. Total lysates were incubated in ice for 30 min and then spun down by centrifugation at 16,000×*g* for 20 min. Total protein concentrations were determined using the BCA method (Thermo Scientific, IL, USA). The extracts were then heated for 5 min in Laemmli loading buffer with 50 mM DTT and 25–50 µg were separated by SDS-PAGE 4–15% gradient gels (Bio-Rad) and transferred to PVDF membranes for immunoblotting. Membranes were blocked with SEA Blocking Buffer (Thermo Scientific, IL, USA) for 1 h. The following primary antibodies were purchased from BD Transduction Labs (CA, USA): anti-utrophin (#610896, 1∶1000), anti-eNOS (#610296, 1∶1000). Anti-CAPON (#ab90854 1∶1000), anti-phospho-(Ser632)-eNOS (#ab76199, 1∶1000) were obtained from ABCAM (MA, USA). Anti-nNOS (#4236S, 1∶1000), anti-IκBα (#4814P, 1∶1000), anti-NF-κB p65 (#8242P, 1∶1000) and GAPDH (#2118S, 1∶5000) were purchased from Cell Signaling (MA, USA). Primary antibodies were diluted in blocking buffer with 0.1% Tween-20, incubated overnight at 4°C, and then washed with TBS 0.1% Tween-20 (TBS-T). Primary antibodies were exposed with either anti-mouse IRDye 680 nm or anti-rabbit IRDye 800 nm antibodies (Li-COR Biosciences, NE, USA), washed, and then quantified with an Odyssey Imaging System (Li-COR Biosciences, NE, USA). Protein levels were normalized to GAPDH expression.

### Resting Sarcolemmal Cation entry

Sarcolemmal Ca^2+^ entry rates were estimated by the rate of dye quench by Mn^2+^ entry in myotubes loaded with 5 µM Fura-2-AM as described previously [26]. Briefly, cells were exited at the isosbestic wavelength for Fura-2 (357/7 nn) and fluorescence emission at 510 nm was then captured from regions of interest within each myotube. Rate of Ca^2+^ entry were estimated from the slope of the quenched signal and expressed as fluorescence arbitrary units per second (f.a.u/s).

### Ethics approval

All procedures for animal experimentation were done in accordance with guidelines approved by the Animal Care and Use Committee (IACUC) at University of California at Davis (Protocol number: #17298), Brigham and Women’s Hospital Harvard Medical School (Protocol number: #23456), University of Pennsylvania (Protocol number: #803950) and Mount Sinai, Medical Center (Protocol number: #14-22-A-04).

### Statistical analysis

All values are expressed as mean ± SEM. Statistical analysis was performed using two-tailed unpaired *t*-test, or one-way analysis of variance coupled with either Tukey’s or Dunnett *t*-test for multiple measurements to determine significance (*P*<0.05).

## Results

### pGz reduces [Ca^2+^]_i_ and [Na^+^]_i_ *in* *vivo* in *mdx* muscles

**Figure 1:**
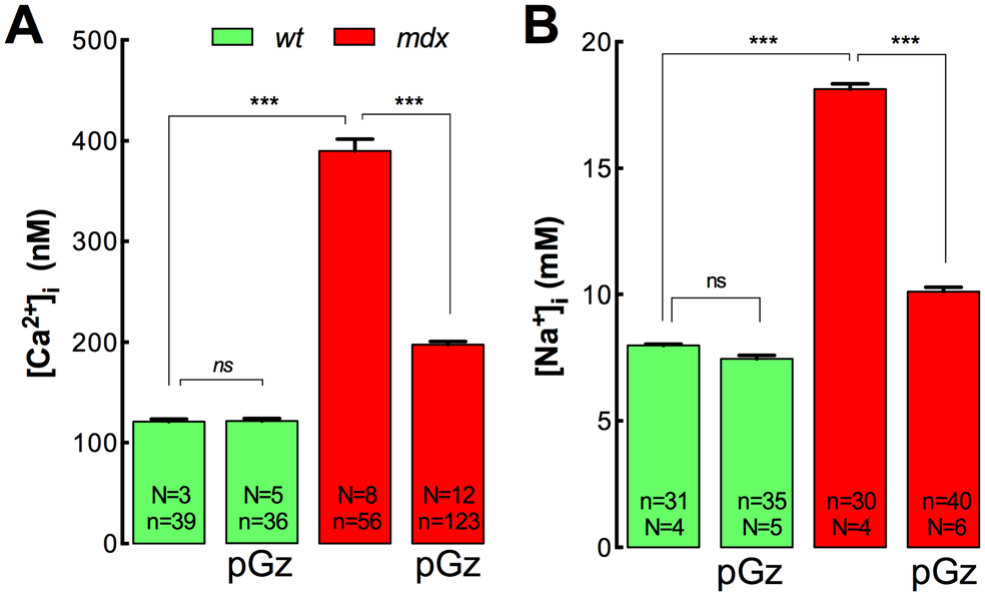
pGz reduces muscle [Ca^2+^]_i_ and [Na^+^]_i_ *in* *vivo* in *mdx* mice. Mice were treated daily for 8 days with pGz (480 cpm, 1 h). Animals were anesthetized and ion concentrations were measured *in* *vivo* in superficial fibers of the *vastus lateralis*. A. [Ca^2+^]_i_ measurements B. [Na^+^]_i_ measurements. Data are expressed as mean ± S.E.M. from n fibers in N mice, ***P<0.001, ANOVA-Tukey’s.

It is well documented that skeletal muscle from *mdx* mice and DMD patients have significantly elevated [Ca^2+^]_i_ [8]–[11], [27] and [Na^+^]_i_ [13], [28], [29]. Since Ca^2+^ and Na^+^ overload are hallmarks of the *mdx* and DMD pathology, we studied the effects of pGz treatment on [Ca^2+^]_i_ and [Na^+^]_i_ measured *in* *vivo* in the superficial fibers of *Vastus lateralis* muscles from both *wt* and *mdx* mice. As showed in Figure 1A, *mdx* muscles have a [Ca^2+^]_i_ that is significantly higher than those of *wt* muscles (390±12 nM *vs* 121±3 nM, *P*<0.001). However, after pGz treatment *mdx* muscle fibers showed a significant reduction in the [Ca^2+^]_i_ to 198±3 nM (*P*<0.001), while no significant effect was observed in *wt* muscles (121±3 nM, *P*>0.05) (Figure 1A). Likewise, [Na^+^]_i_ was significantly elevated in *mdx* muscles compared with *wt* muscles (18.1±0.2 *vs* 8.0±0.1 mM, *P*<0.001) (Figure 1B). pGz treatment significantly reduced [Na^+^]_i_ to 10.1±0.2 mM in dystrophic muscles (*P*<0.001), without any significant effect in *wt* muscles (7.5±0.1 mM, *P*<0.001) (Figure 1B).

### Muscle strength is improved by pGz treatment in *mdx* mice

**Figure 2:**
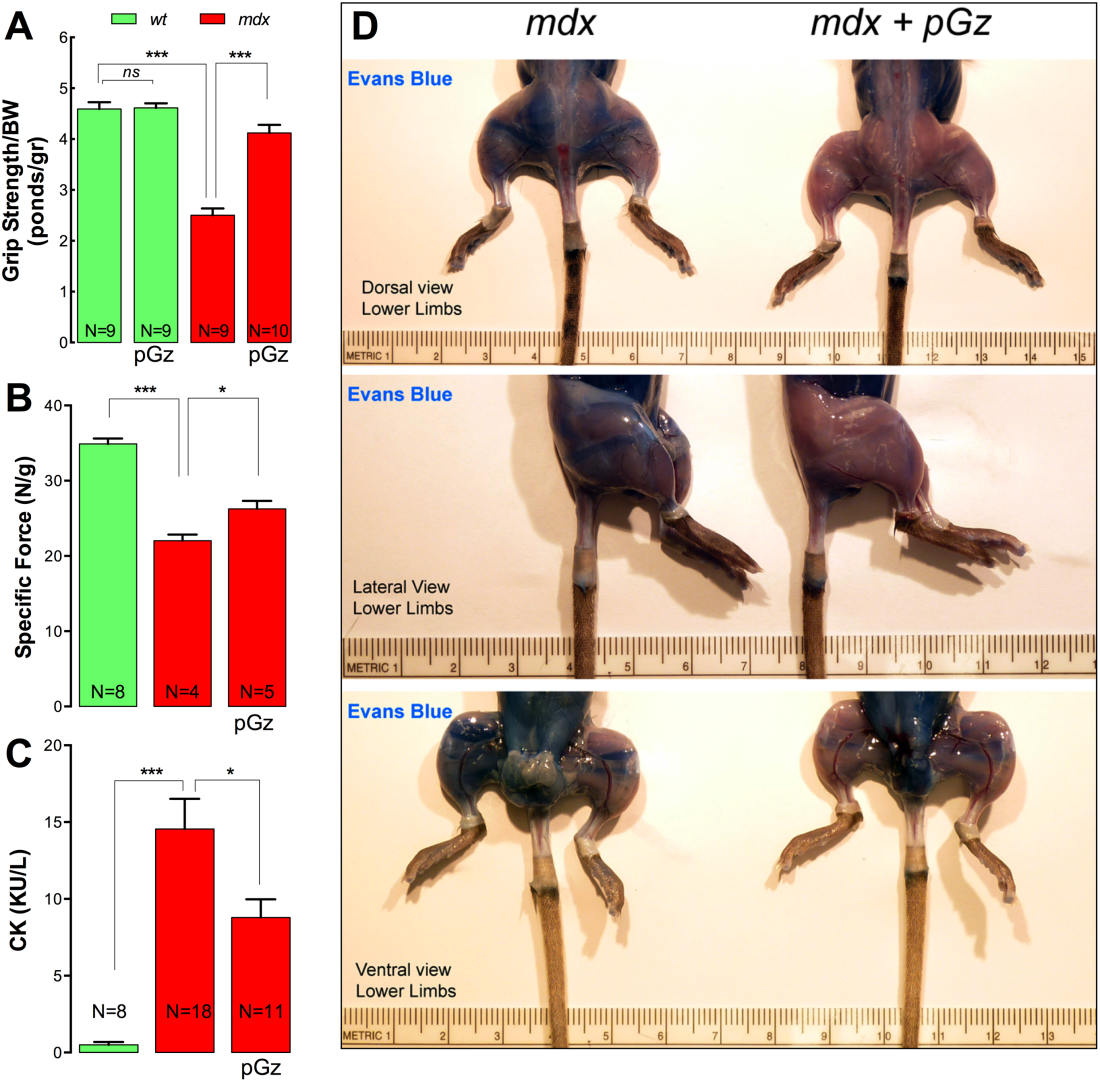
pGz increases muscle strength and reduces muscle damage in *mdx* mice. A. Averaged forelimb grip strength normalized to body weight evaluated after 8 days of pGz treatment. B. *In situ* tetanic specific force measured in *Tibialis Anterior* after 8 days of pGz treatment. C. Serum Creatine Kinase (CK) measurements and D. Evans blue uptake in skeletal muscles from both untreated and pGz-treated *mdx* mice. Data are expressed as mean ± S.E.M. from N mice, *P<0.05 and ***P<0.001, unpaired two-tailed *t*-test.

Both DMD patients and *mdx* models are characterized by muscle weakness as the result of progressive muscle damage [7]. To establish if pGz treatment improves muscle force in dystrophic muscles, forelimb grip strength and tetanic specific force, developed *in* *situ* in *Tibialis Anterior* (TA) muscles, were measured. Comparison of normalized forelimb grip strength values (Figure 2A) revealed that *mdx* mice had a 46% reduction on grip strength compared to *wt* mice (*P*<0.001). After pGz treatment, *mdx* mice had normalized values of forelimb grip strength that were similar to those of untreated *wt* mice (Figure 2A). Similar results were observed in measurements of *in* *situ* tetanic specific force in TA muscles. In untreated *mdx* mice tetanic specific force was significantly decreased in the TA muscles compared with *wt* mice (22.0±0.8 *vs* 34.9±0.7 N/g, *P*<0.001). pGz treatment for 8 days significantly improved TA *in* *situ* tetanic specific force (26.3±1.1 N/g, *P*<0.05 *vs* untreated *mdx* mice) (Figure 2B). These data show that pGz improves muscle function in *mdx* mice, possibly due to a reduction in muscle destruction.

### Reduction of muscle damage by pGz in dystrophic mice

To test the hypothesis that pGz reduces muscle damage in dystrophic muscles serum CK levels and Evans Blue dye (EBD) uptake in skeletal muscles were measured. Figure 2C shows that serum levels of CK in untreated *mdx* mice were significantly elevated in comparison to *wt* mice (14.5±1.9 *vs* 0.5±0.2 KUI/L, *P<*0.001). pGz treatment significantly reduced CK levels in *mdx* mice compared with *mdx* without treatment (8.8±1.2 KUI/L, *P<*0.05) (Figure 2C).

To test for increased sarcolemmal permeability pGz-treated and untreated *mdx* mice were i.p. injected with EBD and euthanized after six hours to obtain dye uptake. EBD is a marker of damaged and permeable muscle fibers in mouse models of muscular dystrophy and as an endpoint in therapeutic trials [30]. Figure 2D shows that *mdx* mice have extensive zones of intense EBD uptake (dark blue staining) in all muscle groups in the ventral, dorsal and lateral views of the hindlimbs. High levels of dye uptake in the abdominal area were observed in both untreated and pGz-treated groups, most likely the result of the i.p. injection of EBD. However, under the same experimental conditions, pGz-treated mice showed much lower levels of EBD uptake in all muscle groups. These results suggest that pGz treatment significantly decreased membrane permeability and muscle damage in *mdx* muscle.

### Expression of Utrophin and CAPON is enhanced by pGz

**Figure 3:**
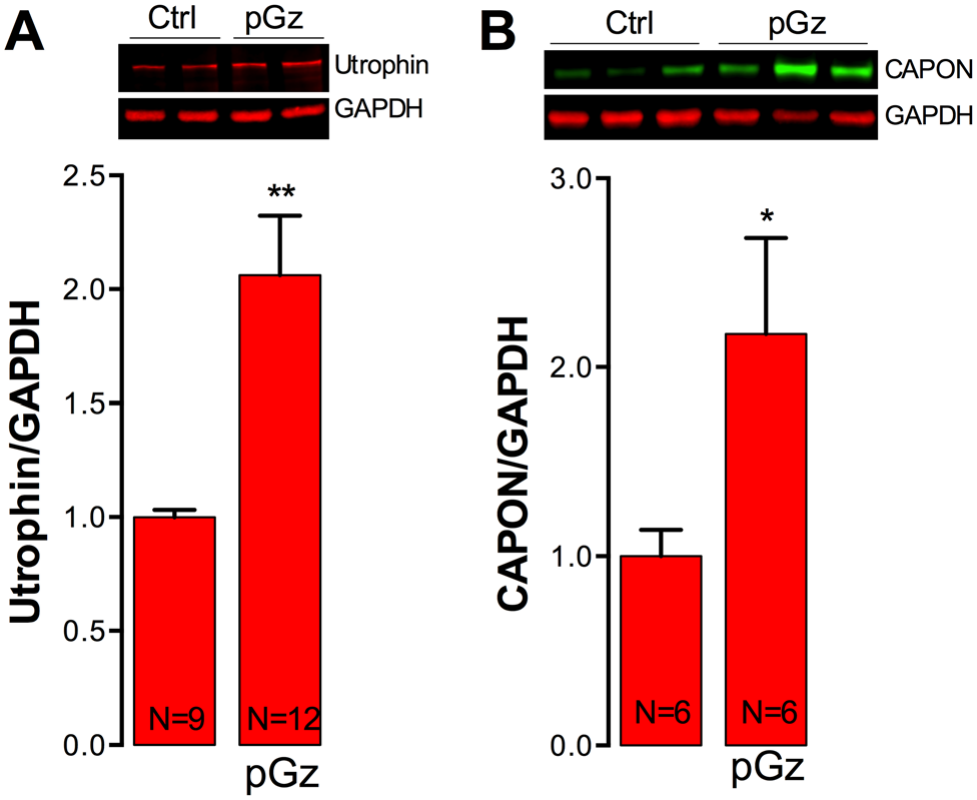
Utrophin and CAPON protein levels in muscles from pGz-treated *mdx* mice. After 8 days of pGz treatment mice were euthanized and the gastrocnemii were dissected and homogenized for western blot analysis. Representative western blots and quantifications of utrophin (A) and CAPON (B) expression. Each lane in the western blot represents a muscle sample obtained from a different mouse. Data were normalized to GAPDH. Data are expressed as mean ± S.E.M. from N mice, *P<0.05 and **P<0.01, unpaired two-tailed *t*-test.

Several studies in the *mdx* mouse model have demonstrated that overexpression of utrophin (an autosomal dystrophin homologue) and/or carboxy-terminal PDZ ligand of nNOS (CAPON) improves dystrophic muscle pathology [31]–[33]. Furthermore, systemic administration of NO precursors has been reported to increase utrophin and CAPON levels in *mdx* muscles [33]–[35]. To determine whether the effect of the pGz treatment observed in dystrophic muscles was associated with up-regulation of utrophin and/or CAPON, the expression of both proteins in gastrocnemius muscles from non-treated and pGz-treated *mdx* mice was measured with Western blot analysis. Figure 3A shows that pGz treatment significantly increased utrophin protein levels by 2.1-fold in *mdx* mice related to untreated-*mdx* mice (*P*<0.01). Likewise, CAPON was significantly up-regulated by 2.2-fold after pGz treatment compared to untreated *mdx* mice (*P*<0.05) (Figure 3B).

### pGz increases IκBα expression in *mdx* muscles

**Figure 4:**
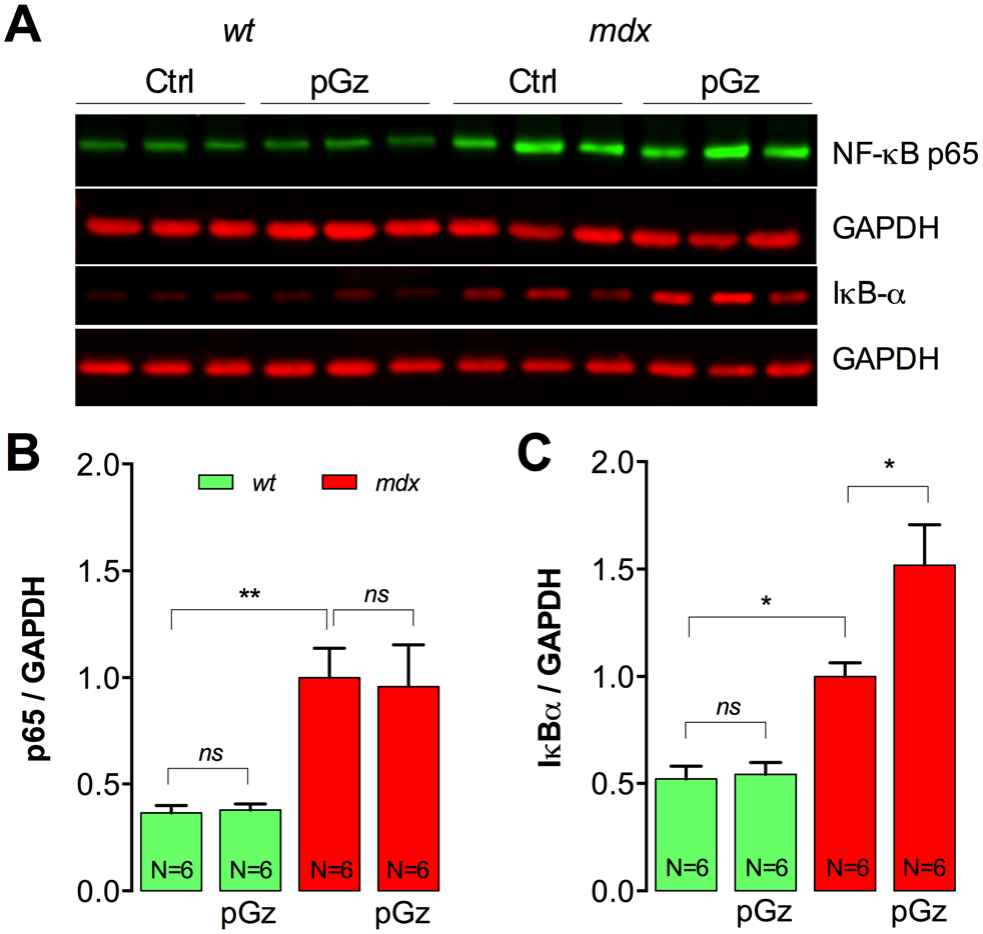
pGz reduces the activation of the NF-κB pro-inflammatory pathway in dystrophic muscles. Total p65 and IκB-α was measured by western blot in gastrocnemii from untreated and pGz-treated mice. A. Representative western blot. B. Quantification of p65 and C. IκB-α. Data were normalized to GAPDH. Each lane in the western blot represents a muscle sample obtained from a different mouse. Data are expressed as mean ± S.E.M. from N mice, *P<0.05 and **P<0.01, ANOVA-Tukey’s.

Treatment with NO precursors, like L-arginine has been shown to reduce the inflammation and the activity of NF-κB as well as an increases in IκB-α expression in *mdx* muscles [35]. We studied protein expression levels of p65 (major NF-κB subunit) and IκB-α (NF-κB repressor) by Western blot in the gastrocnemius of pGz-treated and untreated *wt* and *mdx* mice. There was a 2.7-fold increase in p65 expression and 1.8-fold increase in IκB-α expression in untreated *mdx* muscles compared with *wt* muscles (*P*<0.01) (Figure 4). Whereas pGz did not have any significant effect on p65 expression in either *wt* or *mdx* muscles (*P*>0.05), pGz treatment increased IκB-α by 52% in *mdx* mice (*P*<0.05), without significant change in *wt* muscles (*P*>0.05) (Figure 4). These data suggest a reduction in the inflammatory NF-κB pathway due to a reduction in IκB-α degradation, in dystrophic muscles treated with pGz.

### Decreases in [Ca^2+^]_i_ and [Na^+^]_i_ by pGz in *mdx* myotubes

**Figure 5:**
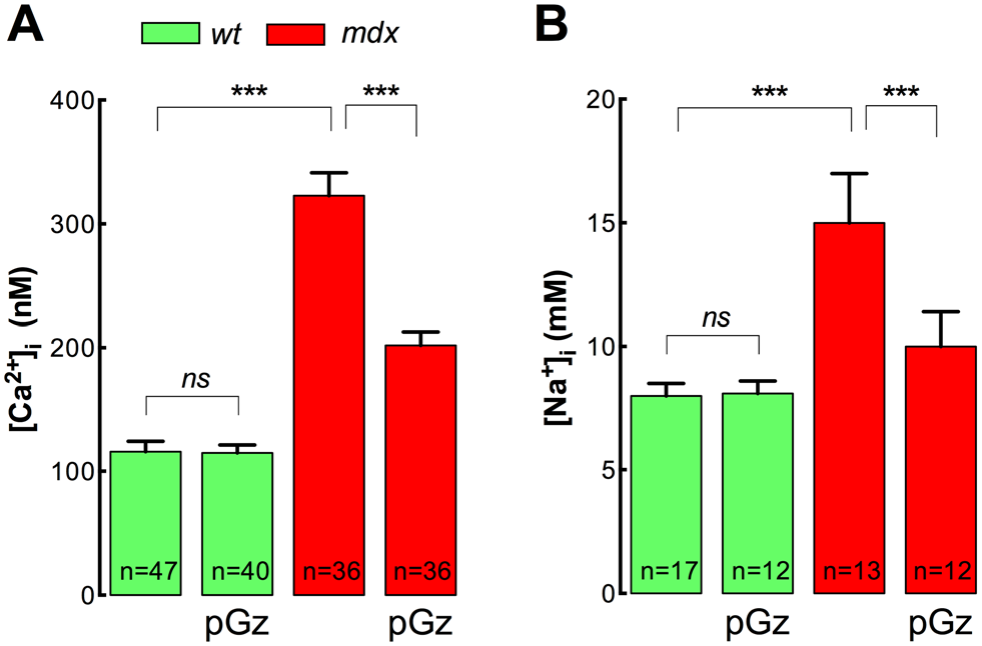
pGz reduces [Ca^2+^]_i_ and [Na^+^]_i_ in *mdx* myotubes. Myotubes were treated with pGz (120 cpm, 12 h) and ion concentrations were measured using double-barreled ion selective microelectrodes. A. [Ca^2+^]_i_ and B. [Na^+^]_i_ determinations. Data are expressed as mean ± S.E.M. ***P<0.001, ANOVA-*Tukey’s.*

Due to the complexity of the multi-systemic response to pGz in dystrophic mice, cultured myotubes were used as an *in* *vitro* model to study the mechanisms of mechano-transduction in skeletal muscle cells. Intracellular ion dysfunction, a hallmark of dystrophic pathology, was taken as an endpoint for the effect of pGz in skeletal muscle cells. Myotubes isolated from *wt* and *mdx* mice were exposed to pGz and then [Ca^2+^]_i_, and [Na^+^]_i_ were measured. Similar to the result in muscle fibers, significant differences in both [Ca^2+^]_i_ and [Na^+^]_i_ were observed between *wt* and *mdx* myotubes (Figure 5). Furthermore, whereas pGz treatment showed no measurable effects on either [Ca^2+^]_i_ (116±1 *vs* 115±1 nM, *P*>0.05) or [Na^+^]_i_ (8.1±0.1 mM *vs* 8.2±0.1 mM *P*>0.05) in *wt* myotubes, it significantly reduced both [Ca^2+^]_i_ (323±3 to 203±2 nM) and in [Na^+^]_i_ (15.7±0.6 to 10.7±0.4 mM) in *mdx* cells (Figure 5). These data reveal that pGz treatment *in* *vitro* provides similar beneficial effects in myotubes to those observed in dystrophic muscles *in* *vivo*, suggesting that pGz effects are mediated by plasma membrane mechanoreceptors under these experimental conditions, and that myotubes are a reliable *in* *vitro* model for the study of the signaling pathways of muscle dystrophy.

### Reduction of resting sarcolemmal Ca^2+^ entry by pGz in *mdx* myotubes

**Figure 6:**
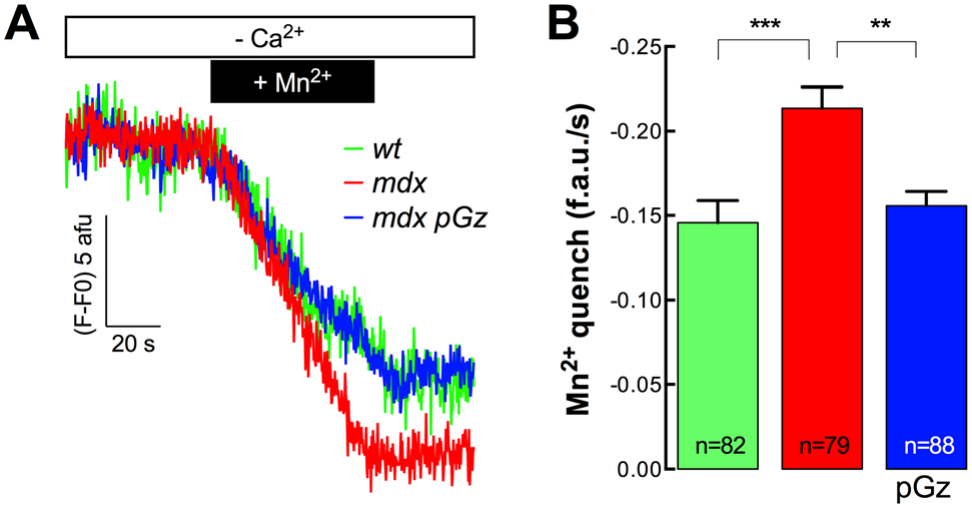
Resting rate of Mn^2+^ quench of Fura-2 fluorescence in pGz-treated *mdx* myotubes. A. Representative traces and B. average resting rates of Mn^2+^ quench. Data are expressed as mean ± S.E.M. **P<0.01, ***P<0.001, ANOVA-*Tukey’s.*

To explore if pGz reduces the abnormal sarcolemmal Ca^2+^ entry that has been observed previously in *mdx* skeletal muscle cells [10], we examined the rate of Fura-2 fluorescence quench by Mn^2+^ to quantify the levels of Ca^2+^ entry in dystrophic myotubes. The Mn^2+^-quench studies indicated that untreated *mdx* myotubes have a significantly increased rate of resting Ca^2+^ entry compared to *wt* myotubes (47% increase, *P*<0.001) and that treatment with pGz was able to reduce resting Ca^2+^ entry to levels similar to that of *wt* (Figure 6). These data demonstrate that pGz treatment decreased the [Ca^2+^]_i_, in part, due to a reduction in the Ca^2+^ entry from the extracellular space in dystrophic myotubes.

### pGz increases eNOS phosphorylation in dystrophic muscle cells

**Figure 7:**
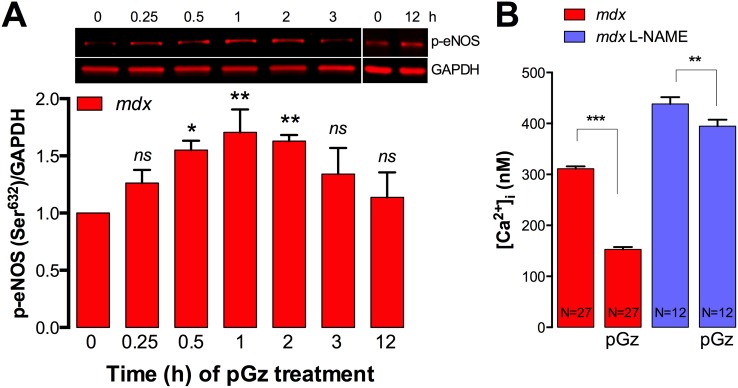
pGz activates eNOS in dystrophic myotubes. A. Myotubes were treated with pGz (120 cpm, 12 h) for the indicated times, quickly lysed in modified RIPA buffer and eNOS phosphorylation (Ser632) was assessed by western blot. B. L-NAME treatment blocks the pGz-induced reduction of [Ca^2+^]_i_ in *mdx* myotubes. Data are expressed as mean ± S.E.M. *P<0.05, **P<0.01, ***P<0.001, ANOVA-*Dunnett’s* (A) and *Tukey’s.* (B).

Beneficial effects of pGz in skeletal muscle have been associated with eNOS activation [36]. Therefore, here we studied the effect of pGz treatment in the time course of eNOS activation in dystrophic myotubes, measured by phosphorylation at Ser632. Cultured *mdx* myotubes were exposed to pGz for increasing amounts of time and then quickly lysed and subjected to Western blot analysis. As showed in Figure 7A, pGz causes a transient increase in eNOS phosphorylation with significant activation after the first 30 min of treatment, reaching a maximum value (1.7±0.2 fold relative to basal levels) after 1 h of treatment, which is followed by a decline to untreated levels by 3 h of treatment.

To further assess the contribution of eNOS on pGz-mediated reduction of [Ca^2+^]_i_, dystrophic myotubes were pre-incubated with L-NAME (1 mM) for 30 min and then treated with pGz. L-NAME was kept in the media for the entire experiment for both the untreated and pGz-treated group. pGz treatment significantly reduced the [Ca^2+^]_i_ from 311±5 to 153±5 in *mdx* myotubes (51% reduction, *P*<0.001), however L-NAME treatment almost completely blunted its ability to lower [Ca^2+^]_i_ (Figure 7B). Since, L-NAME blocks all cNOS, we can not discard the potential participation of nNOS in the pGz-induced [Ca^2+^]_i_ reduction observed in dystrophic myotubes even though its expression in down regulated due to lack of dystrophin.

### Expression of eNOS and nNOS in pGz-treated *mdx* muscles

**Figure 8:**
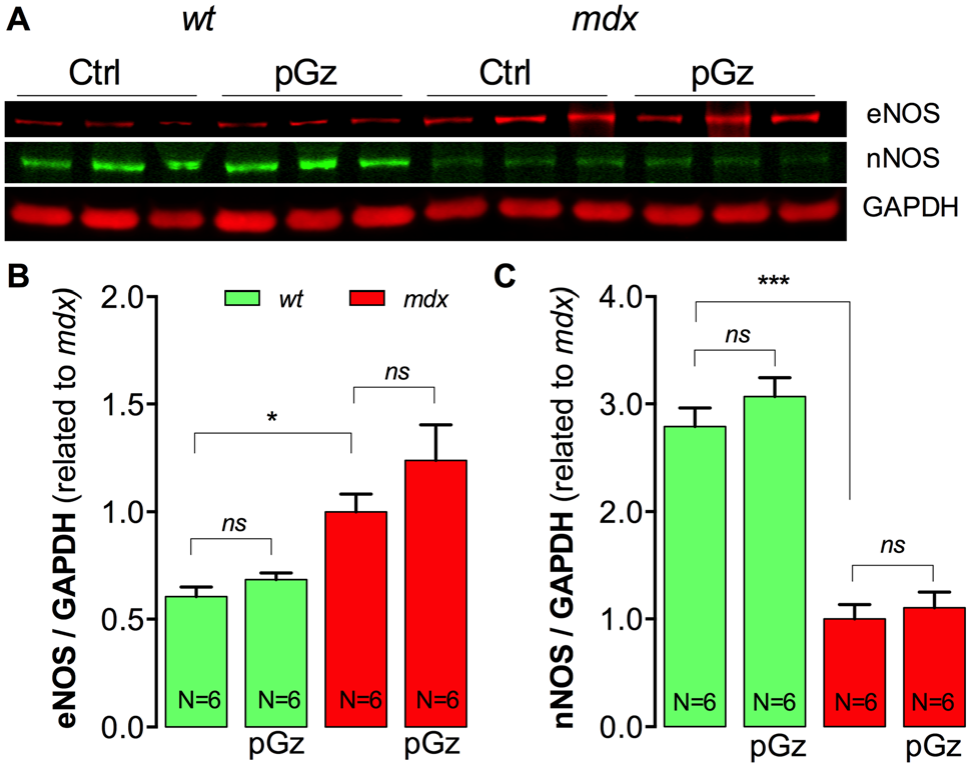
nNOS and eNOS protein levels in muscles from pGz-treated mice. Gastrocnemius were dissected, homogenized and protein expression was assessed by western blot. A. Representative western blot. B. Quantification of eNOS and C. nNOS. Each lane in the western blot represents a muscle sample obtained from a different mouse. Data were normalized with GAPDH. Data are expressed as mean ± S.E.M. from N mice, *P<0.05, and ***P<0.001, ANOVA-Tukey’s.

pGz treatment is associated with an increase in total nNOS and eNOS protein expression in endothelial cells and cardiac muscle [37]. To further study the role of nNOS and eNOS pathways in the effects of pGz on skeletal muscles we studied the expression levels of both proteins in *wt* and *mdx* mice subjected to pGz treatment. Figure 8A shows representative Western blot analysis of eNOS and nNOS expression in *wt* and *mdx* gastrocnemius muscles with and without pGz treatment. Average normalized data indicate that that pGz treatment increased eNOS expression by 24% compared to untreated-*mdx* muscles, but did not reach statistical significance (*P*>0.05, Fig. 8B) without any detectable effect on the expression of nNOS (*P*>0.05, Figure 8C).

## Discussion

Despite of the identification of the molecular defect responsible for DMD several decades ago, there are still no effective cures for the disease. There remains a profound need for alternative therapeutic strategies that can improve muscle strength, ameliorate the dystrophic pathology, and enhance patient quality of life. Here we have examined the benefit of pGz, an enhancer of NO signaling, to treat the *mdx* mouse, a DMD experimental model [38]–[40]. pGz is a non-invasive, drug free approach, which is produced by applying sinusoidal motion to a supine body in a headward-footward direction using a motion platform [39], causing additional pulses to the circulation, increasing pulsatile shear stress to the vascular endothelium and the release of NO [41]. Previous studies have shown that NO therapy has beneficial effects in dystrophic mouse models [22], [34], [35]. Thus, diverse NO-based therapies have been developed to restore intracellular NO homeostasis in *mdx* muscle, like treatment with L-arginine a substrate of nitric oxide synthase for NO synthesis [42], NO donors [43], [44] and more recently to amplify the NO-cGMP signaling pathways with phosphodiesterase (PDE5A) inhibitors. Thus, pGz represent a new and safe therapeutic option for the treatment of DMD based on activation of NO signaling pathway.

We have found that treatment of *mdx* mice with pGz for even a short period of time (8 days) significantly reduced the intracellular Ca^2+^ and Na^+^ overload previously reported by us and others [8], [10], [11], [13], [27] both *in* *vivo* in muscle fibers from *mdx* mice and in cultured *mdx* myotubes. Ca^2+^ and Na^+^ overload have been shown to be deleterious to skeletal muscle fibers, and are associated with either necrotic or apoptotic cell death [29], [45]. These results suggest that the benefits of pGz would not only arise from paracrine effects of the surrounding endothelium but also from direct effects on the muscle cells, possible through activation of mechanoreceptors. Examination of other cell types has also shown that mechanical activity may also be a positive regulator of NOS activity and/or expression and therefore NO production. For example, shear stress applied to endothelial cells *in* *vitro* induces an increase in eNOS mRNA and protein levels [46], [47]. Similarly, compressive loads applied to bones or cyclic strains applied to osteoblasts or osteocytes stimulate NOS activity [48]. Thus, it is possible that outside vessel luminal walls the pGz signaling cascade may be activated through cellular mechanotransduction, mechanism by which cells convert mechanical signals into biochemical responses, increasing physiological NO production by modulating cNOS activity.

In addition, our study indicates that pGz induced a significant reduction in resting sarcolemmal Ca^2+^ entry in cultured *mdx* myotubes. Dystrophic muscle cells are characterized by increased activity of transient receptor potential channels (TRPC), up-regulation of the reverse mode of Na^+^/Ca^2+^ exchanger (NCX1) [13] and hyper-nitrosylation of RyR1 [49] all processes known to either directly or indirectly modulate sarcolemmal Ca^2+^ fluxes. Thus, a reduction in cationic influx induced by pGz tends to restore the intracellular Ca^2+^ and Na^+^ homeostasis in *mdx* muscle cells.

Although, we do not precisely know the mechanisms by which pGz improves Na^+^ and Ca^2+^ homeostasis in *mdx* cells it is possible that this could be linked to the increased expression of utrophin and CAPON. Our experimental findings demonstrated that pGz treatment increased utrophin expression in *mdx* muscles by 2.1-fold which provide membrane stabilization, and CAPON by 2.2-fold which might restore nNOS localization to the sarcolemma, a key criterion for normal NO synthesis in skeletal muscle [33]. Utrophin is slightly smaller than dystrophin (395 kDa) and its primary structure is very similar to that of dystrophin, particularly in the N- and C-terminal ends that bind other proteins. Transgenic overexpression of utrophin in *mdx* mice has been shown to improve muscle function [50], [51]. On the other hand, CAPON expression has been associated to an increased in NOS activity and nNOS expression, previously identified as treatments that reduce disease severity in *mdx* mice [52], [53]. In muscle cells, the localization of nNOS is impaired in the absence of dystrophin, which impacts muscle function, satellite cell activation and NO production [5], [54]. In neurons, the adaptor protein CAPON regulates nNOS localization and activity [55], [56]. CAPON expression has been previously demonstrated in skeletal muscles, and its expression is increased in regenerating skeletal muscle cells [33]. Furthermore, in cardiomyocytes CAPON overexpression induced up-regulation of nNOS and enhanced intracellular NO production, which could both be blocked by treatment with L-NAME [57]. It is very likely that utrophin and CAPON overexpression may have some positive impact on *mdx* muscle cells including a decrease of sarcolemmal Ca^2+^ and restitution of nNOS localization to the sarcolemma. Further experiments need to be carried out to clarify this issue in pGz-treated skeletal muscle cells.

Muscle from dystrophic mice demonstrated muscle weakness compared with muscles from *wt* mice of the same age. Decrements in force generation in dystrophic muscle could have resulted from muscle plasma membrane fragility, impairments in the steps of excitation-contraction coupling, and/or altered contractile protein function in *mdx* muscle [58]. Furthermore, dystrophic skeletal muscle cells have a diminished SR Ca^2+^ loading due to RyR1 and IP_3_R leak at resting conditions [10] and a reduced Ca^2+^ transient elicited by membrane depolarization [59], [60]. All of theses factors may compromise muscle force generation in *mdx* mice. *Mdx* muscles subjected to pGz treatment displayed increased forelimb grip strength and a significant improvement of TA specific force. The study presented here is the first to directly test the ability of pGz to improve skeletal muscle strength in dystrophic mice by performing *in* *vivo* force measurements in the TA. These results are consistent with the hypothesis that a normalization of [Ca^2+^]_i_ by pGz treatment might improved excitation-contraction coupling, decreased muscle damage and/or increased muscle regeneration and thereby force generation in dystrophic muscles. Further research is required to elucidate the mechanisms responsible by which pGz enhances muscle force in *mdx* muscle fibers.

In addition, it has been reported that pGz improves muscle recovery after eccentric exercise in human subjects [61]. Eccentric arm exercises were carried out as 10 sets of 10 lengthening contractions on a seated preacher curl bench using a dumbbell (first day only), followed by 30 min of passive recovery, and 45 min pGz (140 cpm, 45 min per day). pGz treatment significantly increased muscle strength recovery after eccentric exercise, and attenuated CK response even though the reduction was not statistically significant [61].

It is very well established that lack of dystrophin results in fragibility of skeletal muscle fibers, due to loss of muscle fiber membrane integrity, leading to dramatic muscle deterioration [7]. Our studies with EBD in pGz-treated *mdx* mice show a marked reduction of dye incorporation in the majority of the hind limb muscles. Moreover, pGz treatment significantly reduced serum CK activity in *mdx* mice, another way to test plasma membrane integrity, suggesting that it was responsible for a significant reduction in membrane damage and permeability of the dystrophic muscles.

Moreover, pGz increases expression of IκBα (1.5-fold), a member of a family of cellular proteins that function to inhibit the pro-inflammatory NF-κB transcription factor in *mdx* muscle fibers. Similar results have been observed with L-arginine treatment showing a decreased IκBα degradation in *mdx* diaphragm [35]. It has been reported that resting intracellular Ca^2+^ is indirectly implicated in NO production through the modulation of the NF-κB pathway in *mdx* mice [10]. A decrease in [Ca^2+^]_i_ and a restoration of the NF-κB pathway, most likely via regulation of NO intracellular homeostasis would ameliorate muscle destruction in *mdx* muscle fibers.

Although the molecular mechanism by which pGz mitigates muscle damage in *mdx* mice is still unknown, it is likely to involve NO pathways. NO is gaseous messenger that conveys information based on rates of production, localization and concentration [62], [63] and whose protective effect in DMD results from multiple site of actions. Increases in NO signaling has been shown to reduce muscle infiltration [35], [64], decrease intracellular Ca^2+^ release [65], reduce chronic elevation of [Ca^2+^]_i_ in *mdx* cardiomyocytes [66], increase the expression of cytoskeletal proteins in the integrin complex [67] and utrophin and CAPON [33], [42], [65], [68]. In the present study we found that pGz increases eNOS phosphorylation in *mdx* myotubes that peaked after 1 h, returning to a non-significant level after 12 h. These results suggest that salutary effect of pGz appears to be mediated through the NO signaling pathway restoring the intracellular NO homeostasis in *mdx* muscle cells. Moreover, L-NAME treatment blunted the pGz-mediated [Ca^2+^]_i_ reduction in *mdx* myotubes, showing that cNOS plays a role in the downstream signaling pathways.

In summary, in this study we have demonstrated that pGz meets several criteria for promising an effective therapy for DMD. These conclusions are supported by the fact that pGz was efficient in correcting the intracellular ion dyshomeostasis and the enhanced Ca^2+^ entry in *mdx* muscle cells. Most importantly, pGz treatment increased muscle strength measured by forelimb grip strength test and in situ force measurements in TA muscles. Furthermore, pGz treatment significantly decreased muscle injury evidenced by a reduction of Evans Blue incorporation in the limb muscles and a decrease in serum CK levels. This reduction is most likely the result of an increase in mechanical stabilization as a result of an augmented expression in utrophin. Finally, the increase in CAPON in *mdx* muscles may potentially improve intracellular NO production by nNOS restitution to the plasma membrane.
